# 6-Chloro-*N* ^4^,*N* ^4^-dimethyl­pyrimidine-2,4-diamine

**DOI:** 10.1107/S1600536812013517

**Published:** 2012-04-04

**Authors:** Yuan-Yuan Pang, Kai Yu, Bin Sun, Dian-Shun Guo

**Affiliations:** a Department of Chemistry, Shandong Normal University, Jinan 250014, People’s Republic of China

## Abstract

The asymmetric unit of the title compound, C_6_H_9_ClN_4_, contains four independent mol­ecules (*A*, *B*, *C* and *D*). Their main difference is the torsion angles, ranging from 1.6 (5) to 5.9 (5)°, between the methyl group and the pyrimidine plane. A pair of inter­molecular N—H⋯N hydrogen bonds link mol­ecules *A* and *C* into a twisted dimer with a dihedral angle of 32.9 (1)° between the two pyrimidine rings, creating an *R*_2_^2^(8) motif. In the packing, each two mol­ecules of *B*, *C* and *D* form centrosymmetric dimers through two inter­molecular N—H⋯N hydrogen bonds, locally creating *R*_2_^2^(8) motifs. The dimers of *C* and *D* are alternately bridged by *A* into an infinite zigzag strip, locally creating two different *R*_2_^2^(8) motifs with dihedral angles of 32.9 (1) and 63.4 (1)° between the pyrimidine rings. Finally, these strips together with the dimers of *B* associate into a complicated three-dimensional framework.

## Related literature

For background to pyrimidine derivatives, see: Ligthart *et al.* (2005 ▶); Rabie *et al.* (2007 ▶); Goswami *et al.*(2008 ▶); Sherrington & Taskinen (2001 ▶). For similar structures, see: Cetina *et al.* (2005 ▶); Fun *et al.* (2006 ▶); Li *et al.* (2008 ▶); Ebenezer & Muthiah (2010 ▶); Cheng *et al.* (2011 ▶). For hydrogen-bond motifs, see: Bernstein *et al.* (1995 ▶).
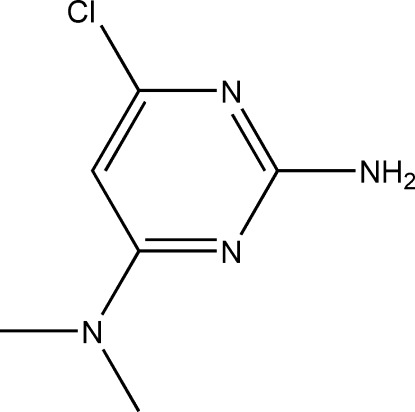


## Experimental

### 

#### Crystal data

C_6_H_9_ClN_4_
*M* *_r_* = 172.62
Triclinic, 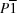

*a* = 7.9438 (19) Å
*b* = 14.225 (3) Å
*c* = 14.895 (3) Å
α = 100.114 (3)°
β = 94.421 (4)°
γ = 100.524 (4)°
*V* = 1618.7 (7) Å^3^
*Z* = 8
Mo *K*α radiation
μ = 0.41 mm^−1^
*T* = 298 K
0.21 × 0.18 × 0.16 mm

#### Data collection

Bruker SMART CCD area-detector diffractometer
Absorption correction: multi-scan (*SADABS*; Bruker, 1999 ▶) *T*_min_ = 0.919, *T*_max_ = 0.937
8239 measured reflections
5619 independent reflections
3769 reflections with *I* > 2σ(*I*)
*R*_int_ = 0.029

#### Refinement

*R*[*F* ^2^ > 2σ(*F* ^2^)] = 0.058
*wR*(*F* ^2^) = 0.136
*S* = 0.97
5619 reflections
405 parameters
H-atom parameters constrained
Δρ_max_ = 0.27 e Å^−3^
Δρ_min_ = −0.32 e Å^−3^

### 

Data collection: *SMART* (Bruker, 1999 ▶); cell refinement: *SAINT* (Bruker, 1999 ▶); data reduction: *SAINT*; program(s) used to solve structure: *SHELXS97* (Sheldrick, 2008 ▶); program(s) used to refine structure: *SHELXL97* (Sheldrick, 2008 ▶); molecular graphics: *SHELXTL* (Sheldrick, 2008 ▶); software used to prepare material for publication: *SHELXTL*.

## Supplementary Material

Crystal structure: contains datablock(s) I, global. DOI: 10.1107/S1600536812013517/zq2159sup1.cif

Structure factors: contains datablock(s) I. DOI: 10.1107/S1600536812013517/zq2159Isup2.hkl

Supplementary material file. DOI: 10.1107/S1600536812013517/zq2159Isup3.cml

Additional supplementary materials:  crystallographic information; 3D view; checkCIF report

## Figures and Tables

**Table 1:** Hydrogen-bond geometry (Å, °)

*D*—H⋯*A*	*D*—H	H⋯*A*	*D*⋯*A*	*D*—H⋯*A*
N11—H11*B*⋯N1	0.87	2.48	3.310 (4)	161
N3—H3*A*⋯N9	0.86	2.41	3.264 (4)	168
N16—H16*B*⋯N15^i^	0.87	2.17	3.038 (4)	174
N11—H11*A*⋯N10^ii^	0.87	2.21	3.086 (4)	174
N7—H7*B*⋯N5^iii^	0.88	2.19	3.064 (3)	178
N3—H3*B*⋯N6^iv^	0.89	2.35	3.230 (4)	169
N7—H7*A*⋯N2^iv^	0.87	2.33	3.136 (3)	155

Symmetry codes: (i) 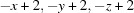; (ii) 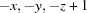; (iii) 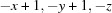; (iv) 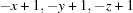.

**Fig. 1.**
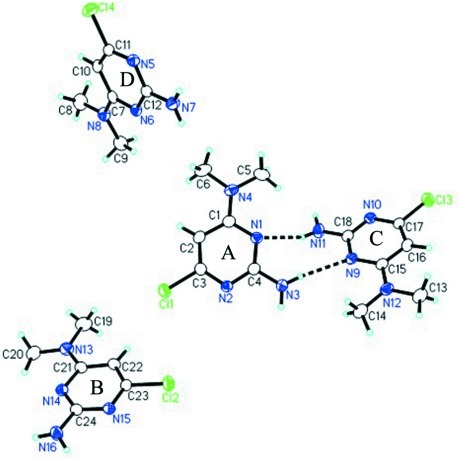
The molecular structure of the title compound, with displacement ellipsoids drawn at the 30% probability level. Dashed lines indicate hydrogen bonds.

**Fig. 2.**
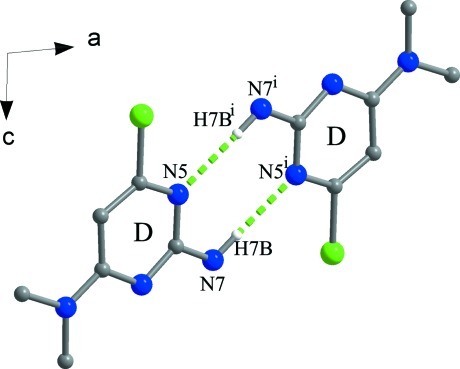
A centrosymmetric dimer of the D molecule, viewed along the crystallographic b axis, showing the R22(8) motif. For the sake of clarity, H atoms not involved in the motif have been omitted [symmetry code: (i) -x + 1, -y + 1, -z].

**Fig. 3.**
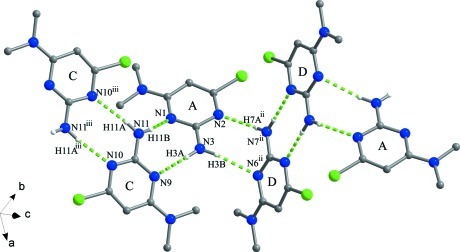
An infinite zigzag strip formed by a combination of A, C and D through hydrogen bonds, showing different R22(8) motifs. For the sake of clarity, H atoms not involved in the motifs have been omitted [symmetry codes: (ii) -x + 1, -y + 1, -z + 1; (iii) -x, -y, -z + 1].

## supplementary crystallographic information

### Comment

Functionalized pyrimidines play a major role in molecular recognition and supramolecular chemistry because of their potential ability to form stable hydrogen-bonded chains *via* their stereochemically associated amino groups and annular N atoms (Sherrington *et al.*, 2001; Ligthart *et al.*, 2005; Rabie *et al.*, 2007; Goswami *et al.*, 2008; Cheng *et al.*, 2011). Lots of substituted 2-aminopyrimidine compounds have been elucidated (Fun *et al.*, 2006; Li *et al.*, 2008; Ebenezer *et al.*, 2010; Cheng *et al.*, 2011). We report here the crystal structure of a related organic compound, 6-chloro-*N*^4^,*N*^4^-dimethylpyrimidine-2,4-diamine.

The title compound, C_6_H_9_ClN_4_, contains four crystallographically independent molecules (Fig. 1), A, B, C and D, in the asymmetric unit, among which A and C are connected into a twisted dimer by two intermolecular N3—H3*A*···N9 and N11—H11*B*···N1 hydrogen bonds (Table 1), with a dihedral angle of 32.9 (1)° between the two pyrimidine rings. The bond lengths and angles of all molecules are similar except for the torsion angles between the methyl groups and the pyrimidine rings, ranging from 1.6 (5) to 5.9 (5)°.

In the packing of the title compound, each inversion-related two molecules of B, C and D form a centrosymmetric dimer through two intermolecular N—H···N hydrogen bonds (Table 1), locally creating an *R*_2_^2^(8) motif (Bernstein *et al.*, 1995). For example, in the dimer of D (Fig. 2), hydrogen bonds arise from atoms N7—H7*B* at (*x*, *y*, *z*) and (-*x* + 1, -*y* + 1, -*z*), which act as hydrogen-bond donors, respectively, to atoms N5 at (-*x* + 1, -*y* + 1, -*z*) and (*x*, *y*, *z*). The dimers of C and D are alternately arranged and bridged by the molecule A, creating an infinite zigzag strip (Cetina *et al.*, 2005) (Fig. 3). In such a strip, the amino group of each molecule acts as a dual donor in N—H···N hydrogen bonds, while the pyrimidine ring serves as a dual acceptor. Interestingly, two different *R*_2_^2^(8) motifs are formed with dihedral angles of 32.9 (1) and 63.4 (1)° between two adjacent pyrimidine rings. Finally, these strips together with the dimers of B are packed into a complicated three dimensional framework.

### Experimental

2-Amino-4,6-dichloropyrimidine (0.082 g, 0.5 mmol) and K_2_CO_3_ (0.276 g, 2 mmol) were dissolved in DMF (2 ml) and H_2_O (2 ml), the mixture was heated at 343 K for 0.5 h and then cooled to room temperature. The resulting mixture was extracted with ethyl acetate. The organic layer was separated and washed with brine, then dried over anhydrous MgSO_4_. Removal of the solvent under reduced pressure gave the title compound as a yellow solid (yield 85%). Single crystals suitable for X-ray diffraction analysis were obtained by liquid–liquid diffusion from hexane and CH_2_Cl_2_ at 298 K.

### Refinement

All non-hydrogen atoms were refined with anisotropic displacement parameters. Hydrogen atoms attached to anisotropically refined atoms were placed in geometrically idealized positions and included as riding atoms with C—H = 0.93 Å and *U*_iso_(H) = 1.2 *U*_eq_(C) (aromatic); C—H = 0.96 Å and *U*_iso_(H) = 1.5 *U*_eq_(C) (methyl); N—H = 0.87–0.88 Å and *U*_iso_(H) = 1.2*U*_eq_(N).

### Crystal data

**Table:**

C_6_H_9_ClN_4_	*Z* = 8
*M**_r_* = 172.62	*F*(000) = 720
Triclinic, *P*1	*D*_x_ = 1.417 Mg m^−^^3^
Hall symbol: -P 1	Mo *K*α radiation, λ = 0.71073 Å
*a* = 7.9438 (19) Å	Cell parameters from 2311 reflections
*b* = 14.225 (3) Å	θ = 2.6–27.7°
*c* = 14.895 (3) Å	µ = 0.41 mm^−^^1^
α = 100.114 (3)°	*T* = 298 K
β = 94.421 (4)°	Block, colourless
γ = 100.524 (4)°	0.21 × 0.18 × 0.16 mm
*V* = 1618.7 (7) Å^3^	

### Data collection

**Table:**

Bruker SMART CCD area-detector diffractometer	5619 independent reflections
Radiation source: fine-focus sealed tube	3769 reflections with *I* > 2σ(*I*)
Graphite monochromator	*R*_int_ = 0.029
phi and ω scans	θ_max_ = 25.0°, θ_min_ = 1.5°
Absorption correction: multi-scan (*SADABS*; Bruker, 1999)	*h* = −9→8
*T*_min_ = 0.919, *T*_max_ = 0.937	*k* = −16→16
8239 measured reflections	*l* = −17→15

### Refinement

**Table:**

Refinement on *F*^2^	Primary atom site location: structure-invariant direct methods
Least-squares matrix: full	Secondary atom site location: difference Fourier map
*R*[*F*^2^ > 2σ(*F*^2^)] = 0.058	Hydrogen site location: inferred from neighbouring sites
*wR*(*F*^2^) = 0.136	H-atom parameters constrained
*S* = 0.97	*w* = 1/[σ^2^(*F*_o_^2^) + (0.0681*P*)^2^] where *P* = (*F*_o_^2^ + 2*F*_c_^2^)/3
5619 reflections	(Δ/σ)_max_ = 0.001
405 parameters	Δρ_max_ = 0.27 e Å^−^^3^
0 restraints	Δρ_min_ = −0.32 e Å^−^^3^

### Special details

**Table:**

Geometry. All e.s.d.'s (except the e.s.d. in the dihedral angle between two l.s. planes) are estimated using the full covariance matrix. The cell e.s.d.'s are taken into account individually in the estimation of e.s.d.'s in distances, angles and torsion angles; correlations between e.s.d.'s in cell parameters are only used when they are defined by crystal symmetry. An approximate (isotropic) treatment of cell e.s.d.'s is used for estimating e.s.d.'s involving l.s. planes.
Refinement. Refinement of *F*^2^ against ALL reflections. The weighted *R*-factor *wR* and goodness of fit *S* are based on *F*^2^, conventional *R*-factors *R* are based on *F*, with *F* set to zero for negative *F*^2^. The threshold expression of *F*^2^ > σ(*F*^2^) is used only for calculating *R*-factors(gt) *etc*. and is not relevant to the choice of reflections for refinement. *R*-factors based on *F*^2^ are statistically about twice as large as those based on *F*, and *R*- factors based on ALL data will be even larger.

### Fractional atomic coordinates and isotropic or equivalent isotropic displacement parameters (Å^2^)

**Table:**

	*x*	*y*	*z*	*U*_iso_*/*U*_eq_	
N1	0.3407 (3)	0.34115 (18)	0.54662 (16)	0.0383 (6)	
N2	0.4648 (3)	0.47960 (18)	0.66542 (16)	0.0369 (6)	
N3	0.5150 (4)	0.32771 (19)	0.67110 (18)	0.0534 (8)	
N4	0.1803 (4)	0.3573 (2)	0.41724 (17)	0.0498 (8)	
N5	0.3131 (3)	0.56029 (17)	0.00737 (15)	0.0377 (6)	
N6	0.2031 (3)	0.57494 (17)	0.15383 (16)	0.0356 (6)	
N7	0.4281 (4)	0.49791 (19)	0.12364 (16)	0.0469 (8)	
N8	−0.0361 (4)	0.64285 (19)	0.18002 (18)	0.0451 (7)	
N9	0.4193 (3)	0.08990 (18)	0.62696 (16)	0.0379 (6)	
N10	0.1931 (3)	−0.03793 (18)	0.54295 (16)	0.0390 (7)	
N11	0.1596 (4)	0.12009 (19)	0.5723 (2)	0.0542 (8)	
N12	0.6743 (4)	0.0572 (2)	0.68555 (18)	0.0478 (7)	
N13	0.3235 (4)	0.9764 (2)	0.83692 (19)	0.0511 (8)	
N14	0.5994 (4)	1.03959 (18)	0.90517 (17)	0.0405 (7)	
N15	0.8008 (3)	0.93718 (19)	0.93227 (17)	0.0412 (7)	
N16	0.8765 (4)	1.10315 (19)	0.9711 (2)	0.0567 (8)	
C1	0.2689 (4)	0.3996 (2)	0.4992 (2)	0.0387 (8)	
C2	0.2883 (4)	0.4998 (2)	0.5353 (2)	0.0405 (8)	
H2	0.2382	0.5412	0.5047	0.049*	
C3	0.3852 (4)	0.5320 (2)	0.6179 (2)	0.0370 (8)	
C4	0.4379 (4)	0.3849 (2)	0.6250 (2)	0.0399 (8)	
C5	0.1705 (5)	0.2558 (3)	0.3791 (2)	0.0619 (11)	
H5A	0.2531	0.2309	0.4139	0.093*	
H5B	0.1951	0.2490	0.3164	0.093*	
H5C	0.0568	0.2198	0.3815	0.093*	
C6	0.1034 (5)	0.4146 (3)	0.3586 (2)	0.0667 (11)	
H6A	0.0397	0.4559	0.3942	0.100*	
H6B	0.0272	0.3715	0.3092	0.100*	
H6C	0.1929	0.4541	0.3340	0.100*	
C7	0.0773 (4)	0.6171 (2)	0.1222 (2)	0.0362 (8)	
C8	−0.1760 (5)	0.6879 (3)	0.1505 (3)	0.0643 (11)	
H8A	−0.1297	0.7441	0.1262	0.096*	
H8B	−0.2361	0.7072	0.2020	0.096*	
H8C	−0.2544	0.6420	0.1039	0.096*	
C9	−0.0343 (5)	0.6179 (3)	0.2706 (2)	0.0570 (10)	
H9A	−0.1284	0.5649	0.2705	0.086*	
H9B	−0.0458	0.6735	0.3150	0.086*	
H9C	0.0725	0.5990	0.2861	0.086*	
C10	0.0687 (4)	0.6347 (2)	0.0317 (2)	0.0376 (8)	
H10	−0.0150	0.6648	0.0084	0.045*	
C11	0.1901 (4)	0.6050 (2)	−0.01859 (19)	0.0363 (8)	
C12	0.3096 (4)	0.5455 (2)	0.09442 (19)	0.0348 (7)	
C13	0.7969 (5)	−0.0067 (3)	0.6925 (2)	0.0623 (11)	
H13A	0.7474	−0.0597	0.7204	0.093*	
H13B	0.9006	0.0295	0.7293	0.093*	
H13C	0.8232	−0.0321	0.6322	0.093*	
C14	0.7279 (5)	0.1570 (2)	0.7337 (3)	0.0610 (11)	
H14A	0.7736	0.1970	0.6921	0.092*	
H14B	0.8149	0.1607	0.7833	0.092*	
H14C	0.6306	0.1797	0.7577	0.092*	
C15	0.5186 (4)	0.0239 (2)	0.63617 (19)	0.0375 (8)	
C16	0.4614 (4)	−0.0744 (2)	0.5956 (2)	0.0418 (8)	
H16	0.5303	−0.1203	0.5987	0.050*	
C17	0.2997 (4)	−0.0987 (2)	0.55161 (19)	0.0367 (8)	
C18	0.2619 (4)	0.0556 (2)	0.5810 (2)	0.0380 (8)	
C19	0.1863 (5)	0.8969 (3)	0.7904 (3)	0.0694 (12)	
H19A	0.2290	0.8584	0.7410	0.104*	
H19B	0.0928	0.9228	0.7663	0.104*	
H19C	0.1462	0.8569	0.8330	0.104*	
C20	0.2873 (5)	1.0738 (3)	0.8461 (2)	0.0594 (11)	
H20A	0.3797	1.1194	0.8848	0.089*	
H20B	0.1816	1.0755	0.8730	0.089*	
H20C	0.2765	1.0909	0.7867	0.089*	
C21	0.4788 (4)	0.9608 (2)	0.8677 (2)	0.0394 (8)	
C22	0.5135 (4)	0.8663 (2)	0.8614 (2)	0.0447 (9)	
H22	0.4315	0.8106	0.8359	0.054*	
C23	0.6750 (5)	0.8624 (2)	0.8952 (2)	0.0419 (8)	
C24	0.7528 (4)	1.0241 (2)	0.9355 (2)	0.0396 (8)	
Cl1	0.41068 (13)	0.65460 (6)	0.66924 (6)	0.0559 (3)	
Cl2	0.72990 (15)	0.74846 (7)	0.89197 (7)	0.0725 (3)	
Cl3	0.21621 (13)	−0.21962 (6)	0.49962 (6)	0.0594 (3)	
Cl4	0.19291 (13)	0.62621 (7)	−0.13080 (5)	0.0581 (3)	
H7A	0.4312	0.4882	0.1794	0.070*	
H11B	0.2061	0.1818	0.5803	0.070*	
H16A	0.8516	1.1614	0.9822	0.070*	
H3A	0.4991	0.2653	0.6522	0.070*	
H7B	0.5017	0.4822	0.0852	0.070*	
H16B	0.9661	1.0935	1.0027	0.070*	
H3B	0.5805	0.3546	0.7235	0.070*	
H11A	0.0579	0.1009	0.5408	0.070*	

### Atomic displacement parameters (Å^2^)

**Table:**

	*U*^11^	*U*^22^	*U*^33^	*U*^12^	*U*^13^	*U*^23^
N1	0.0343 (17)	0.0405 (16)	0.0382 (14)	0.0061 (13)	−0.0005 (12)	0.0055 (12)
N2	0.0337 (17)	0.0400 (16)	0.0369 (14)	0.0079 (13)	0.0031 (12)	0.0067 (12)
N3	0.066 (2)	0.0450 (17)	0.0496 (17)	0.0224 (16)	−0.0095 (15)	0.0045 (13)
N4	0.047 (2)	0.060 (2)	0.0405 (16)	0.0082 (15)	−0.0063 (14)	0.0101 (14)
N5	0.0362 (17)	0.0406 (16)	0.0368 (15)	0.0086 (13)	0.0070 (12)	0.0070 (11)
N6	0.0336 (17)	0.0365 (15)	0.0388 (14)	0.0103 (13)	0.0078 (12)	0.0078 (11)
N7	0.050 (2)	0.0593 (19)	0.0398 (15)	0.0284 (16)	0.0106 (13)	0.0122 (13)
N8	0.0385 (18)	0.0476 (17)	0.0540 (17)	0.0158 (14)	0.0164 (14)	0.0101 (13)
N9	0.0298 (16)	0.0432 (16)	0.0411 (15)	0.0069 (13)	−0.0009 (12)	0.0115 (12)
N10	0.0314 (17)	0.0422 (17)	0.0415 (15)	0.0040 (14)	−0.0016 (12)	0.0093 (12)
N11	0.0368 (19)	0.0418 (17)	0.081 (2)	0.0086 (14)	−0.0111 (16)	0.0110 (14)
N12	0.0370 (18)	0.0517 (19)	0.0523 (17)	0.0066 (15)	−0.0087 (14)	0.0122 (14)
N13	0.0315 (18)	0.059 (2)	0.0599 (18)	0.0097 (15)	−0.0050 (14)	0.0063 (14)
N14	0.0359 (18)	0.0409 (16)	0.0439 (15)	0.0071 (14)	0.0011 (13)	0.0086 (12)
N15	0.0354 (17)	0.0395 (17)	0.0452 (15)	0.0040 (14)	−0.0049 (13)	0.0062 (12)
N16	0.048 (2)	0.0376 (17)	0.077 (2)	0.0010 (15)	−0.0156 (16)	0.0083 (14)
C1	0.0256 (19)	0.049 (2)	0.0412 (18)	0.0038 (16)	0.0070 (15)	0.0107 (15)
C2	0.033 (2)	0.050 (2)	0.0437 (19)	0.0134 (16)	0.0066 (15)	0.0154 (15)
C3	0.032 (2)	0.0403 (19)	0.0410 (18)	0.0072 (16)	0.0159 (15)	0.0094 (14)
C4	0.033 (2)	0.046 (2)	0.0418 (18)	0.0081 (16)	0.0063 (15)	0.0116 (16)
C5	0.061 (3)	0.060 (3)	0.050 (2)	−0.008 (2)	−0.0051 (19)	−0.0023 (17)
C6	0.054 (3)	0.095 (3)	0.051 (2)	0.019 (2)	−0.0105 (19)	0.017 (2)
C7	0.034 (2)	0.0230 (17)	0.0479 (18)	0.0003 (15)	0.0042 (15)	0.0028 (13)
C8	0.047 (3)	0.069 (3)	0.086 (3)	0.029 (2)	0.020 (2)	0.015 (2)
C9	0.057 (3)	0.059 (2)	0.058 (2)	0.010 (2)	0.0266 (19)	0.0089 (17)
C10	0.035 (2)	0.0297 (18)	0.0488 (19)	0.0062 (15)	0.0008 (16)	0.0107 (14)
C11	0.039 (2)	0.0290 (17)	0.0374 (17)	−0.0008 (15)	0.0005 (15)	0.0074 (13)
C12	0.033 (2)	0.0321 (18)	0.0383 (17)	0.0067 (15)	0.0053 (15)	0.0040 (13)
C13	0.041 (2)	0.076 (3)	0.071 (3)	0.018 (2)	−0.009 (2)	0.017 (2)
C14	0.050 (3)	0.053 (2)	0.073 (3)	−0.0062 (19)	−0.019 (2)	0.0236 (19)
C15	0.031 (2)	0.047 (2)	0.0357 (17)	0.0060 (16)	0.0044 (15)	0.0143 (14)
C16	0.033 (2)	0.048 (2)	0.0485 (19)	0.0157 (17)	0.0056 (16)	0.0128 (16)
C17	0.034 (2)	0.0422 (19)	0.0354 (17)	0.0076 (16)	0.0033 (15)	0.0108 (14)
C18	0.030 (2)	0.046 (2)	0.0405 (17)	0.0097 (17)	0.0055 (15)	0.0134 (15)
C19	0.038 (2)	0.088 (3)	0.076 (3)	0.006 (2)	−0.010 (2)	0.014 (2)
C20	0.052 (3)	0.074 (3)	0.061 (2)	0.032 (2)	0.0055 (19)	0.0148 (19)
C21	0.035 (2)	0.043 (2)	0.0380 (17)	0.0033 (17)	0.0050 (15)	0.0064 (14)
C22	0.039 (2)	0.042 (2)	0.0471 (19)	−0.0002 (17)	−0.0058 (16)	0.0035 (15)
C23	0.046 (2)	0.037 (2)	0.0430 (18)	0.0104 (17)	−0.0022 (17)	0.0085 (14)
C24	0.038 (2)	0.039 (2)	0.0386 (17)	0.0002 (17)	0.0019 (15)	0.0080 (14)
Cl1	0.0686 (7)	0.0406 (5)	0.0582 (5)	0.0122 (5)	0.0097 (5)	0.0062 (4)
Cl2	0.0785 (8)	0.0406 (6)	0.0913 (7)	0.0163 (5)	−0.0270 (6)	0.0051 (5)
Cl3	0.0610 (7)	0.0407 (5)	0.0700 (6)	0.0063 (5)	−0.0074 (5)	0.0037 (4)
Cl4	0.0674 (7)	0.0670 (6)	0.0436 (5)	0.0108 (5)	0.0059 (4)	0.0233 (4)

### Geometric parameters (Å, º)

**Table:**

N1—C4	1.333 (4)	C1—C2	1.409 (4)
N1—C1	1.353 (4)	C2—C3	1.357 (4)
N2—C3	1.321 (4)	C2—H2	0.9300
N2—C4	1.347 (4)	C3—Cl1	1.746 (3)
N3—C4	1.350 (4)	C5—H5A	0.9600
N3—H3A	0.8649	C5—H5B	0.9600
N3—H3B	0.8870	C5—H5C	0.9600
N4—C1	1.343 (4)	C6—H6A	0.9600
N4—C5	1.441 (4)	C6—H6B	0.9600
N4—C6	1.467 (4)	C6—H6C	0.9600
N5—C11	1.329 (4)	C7—C10	1.411 (4)
N5—C12	1.351 (3)	C8—H8A	0.9600
N6—C12	1.338 (4)	C8—H8B	0.9600
N6—C7	1.351 (4)	C8—H8C	0.9600
N7—C12	1.344 (4)	C9—H9A	0.9600
N7—H7A	0.8654	C9—H9B	0.9600
N7—H7B	0.8789	C9—H9C	0.9600
N8—C7	1.348 (4)	C10—C11	1.349 (4)
N8—C9	1.453 (4)	C10—H10	0.9300
N8—C8	1.456 (4)	C11—Cl4	1.751 (3)
N9—C18	1.341 (4)	C13—H13A	0.9600
N9—C15	1.350 (4)	C13—H13B	0.9600
N10—C17	1.330 (4)	C13—H13C	0.9600
N10—C18	1.346 (4)	C14—H14A	0.9600
N11—C18	1.348 (4)	C14—H14B	0.9600
N11—H11B	0.8716	C14—H14C	0.9600
N11—H11A	0.8741	C15—C16	1.399 (4)
N12—C15	1.349 (4)	C16—C17	1.352 (4)
N12—C14	1.444 (4)	C16—H16	0.9300
N12—C13	1.458 (4)	C17—Cl3	1.742 (3)
N13—C21	1.353 (4)	C19—H19A	0.9600
N13—C20	1.450 (4)	C19—H19B	0.9600
N13—C19	1.452 (5)	C19—H19C	0.9600
N14—C24	1.337 (4)	C20—H20A	0.9600
N14—C21	1.338 (4)	C20—H20B	0.9600
N15—C23	1.325 (4)	C20—H20C	0.9600
N15—C24	1.353 (4)	C21—C22	1.409 (4)
N16—C24	1.348 (4)	C22—C23	1.356 (4)
N16—H16A	0.8773	C22—H22	0.9300
N16—H16B	0.8682	C23—Cl2	1.747 (3)
			
C4—N1—C1	116.3 (3)	H8B—C8—H8C	109.5
C3—N2—C4	113.1 (3)	N8—C9—H9A	109.5
C4—N3—H3A	122.0	N8—C9—H9B	109.5
C4—N3—H3B	118.9	H9A—C9—H9B	109.5
H3A—N3—H3B	119.1	N8—C9—H9C	109.5
C1—N4—C5	121.6 (3)	H9A—C9—H9C	109.5
C1—N4—C6	121.2 (3)	H9B—C9—H9C	109.5
C5—N4—C6	116.9 (3)	C11—C10—C7	115.4 (3)
C11—N5—C12	113.1 (3)	C11—C10—H10	122.3
C12—N6—C7	117.2 (2)	C7—C10—H10	122.3
C12—N7—H7A	118.4	N5—C11—C10	127.1 (3)
C12—N7—H7B	116.9	N5—C11—Cl4	114.1 (2)
H7A—N7—H7B	124.6	C10—C11—Cl4	118.8 (2)
C7—N8—C9	121.4 (3)	N6—C12—N7	117.7 (3)
C7—N8—C8	121.0 (3)	N6—C12—N5	126.5 (3)
C9—N8—C8	117.3 (3)	N7—C12—N5	115.8 (3)
C18—N9—C15	116.7 (3)	N12—C13—H13A	109.5
C17—N10—C18	113.7 (3)	N12—C13—H13B	109.5
C18—N11—H11B	119.1	H13A—C13—H13B	109.5
C18—N11—H11A	120.3	N12—C13—H13C	109.5
H11B—N11—H11A	117.1	H13A—C13—H13C	109.5
C15—N12—C14	122.1 (3)	H13B—C13—H13C	109.5
C15—N12—C13	121.0 (3)	N12—C14—H14A	109.5
C14—N12—C13	116.9 (3)	N12—C14—H14B	109.5
C21—N13—C20	121.8 (3)	H14A—C14—H14B	109.5
C21—N13—C19	121.7 (3)	N12—C14—H14C	109.5
C20—N13—C19	116.5 (3)	H14A—C14—H14C	109.5
C24—N14—C21	116.9 (3)	H14B—C14—H14C	109.5
C23—N15—C24	112.7 (3)	N12—C15—N9	117.0 (3)
C24—N16—H16A	120.6	N12—C15—C16	122.0 (3)
C24—N16—H16B	116.8	N9—C15—C16	120.9 (3)
H16A—N16—H16B	117.8	C17—C16—C15	115.9 (3)
N4—C1—N1	116.8 (3)	C17—C16—H16	122.1
N4—C1—C2	122.5 (3)	C15—C16—H16	122.1
N1—C1—C2	120.7 (3)	N10—C17—C16	126.1 (3)
C3—C2—C1	115.4 (3)	N10—C17—Cl3	114.6 (2)
C3—C2—H2	122.3	C16—C17—Cl3	119.3 (2)
C1—C2—H2	122.3	N9—C18—N10	126.6 (3)
N2—C3—C2	126.7 (3)	N9—C18—N11	117.6 (3)
N2—C3—Cl1	114.8 (2)	N10—C18—N11	115.8 (3)
C2—C3—Cl1	118.5 (2)	N13—C19—H19A	109.5
N1—C4—N2	127.6 (3)	N13—C19—H19B	109.5
N1—C4—N3	116.7 (3)	H19A—C19—H19B	109.5
N2—C4—N3	115.7 (3)	N13—C19—H19C	109.5
N4—C5—H5A	109.5	H19A—C19—H19C	109.5
N4—C5—H5B	109.5	H19B—C19—H19C	109.5
H5A—C5—H5B	109.5	N13—C20—H20A	109.5
N4—C5—H5C	109.5	N13—C20—H20B	109.5
H5A—C5—H5C	109.5	H20A—C20—H20B	109.5
H5B—C5—H5C	109.5	N13—C20—H20C	109.5
N4—C6—H6A	109.5	H20A—C20—H20C	109.5
N4—C6—H6B	109.5	H20B—C20—H20C	109.5
H6A—C6—H6B	109.5	N14—C21—N13	116.9 (3)
N4—C6—H6C	109.5	N14—C21—C22	121.0 (3)
H6A—C6—H6C	109.5	N13—C21—C22	122.1 (3)
H6B—C6—H6C	109.5	C23—C22—C21	115.3 (3)
N8—C7—N6	117.9 (3)	C23—C22—H22	122.4
N8—C7—C10	121.8 (3)	C21—C22—H22	122.4
N6—C7—C10	120.4 (3)	N15—C23—C22	126.9 (3)
N8—C8—H8A	109.5	N15—C23—Cl2	114.3 (2)
N8—C8—H8B	109.5	C22—C23—Cl2	118.8 (3)
H8A—C8—H8B	109.5	N14—C24—N16	117.3 (3)
N8—C8—H8C	109.5	N14—C24—N15	127.2 (3)
H8A—C8—H8C	109.5	N16—C24—N15	115.5 (3)
			
C5—N4—C1—N1	−3.6 (5)	C14—N12—C15—N9	5.5 (5)
C6—N4—C1—N1	−177.5 (3)	C13—N12—C15—N9	−174.5 (3)
C5—N4—C1—C2	176.3 (3)	C14—N12—C15—C16	−174.8 (3)
C6—N4—C1—C2	2.4 (5)	C13—N12—C15—C16	5.3 (5)
C4—N1—C1—N4	175.7 (3)	C18—N9—C15—N12	−177.3 (3)
C4—N1—C1—C2	−4.2 (4)	C18—N9—C15—C16	2.9 (4)
N4—C1—C2—C3	−178.4 (3)	N12—C15—C16—C17	177.1 (3)
N1—C1—C2—C3	1.4 (5)	N9—C15—C16—C17	−3.2 (5)
C4—N2—C3—C2	−1.8 (5)	C18—N10—C17—C16	1.8 (5)
C4—N2—C3—Cl1	178.1 (2)	C18—N10—C17—Cl3	−178.0 (2)
C1—C2—C3—N2	1.8 (5)	C15—C16—C17—N10	0.8 (5)
C1—C2—C3—Cl1	−178.2 (2)	C15—C16—C17—Cl3	−179.5 (2)
C1—N1—C4—N2	4.4 (5)	C15—N9—C18—N10	−0.2 (5)
C1—N1—C4—N3	−177.7 (3)	C15—N9—C18—N11	179.0 (3)
C3—N2—C4—N1	−1.5 (5)	C17—N10—C18—N9	−2.1 (5)
C3—N2—C4—N3	−179.4 (3)	C17—N10—C18—N11	178.7 (3)
C9—N8—C7—N6	−5.8 (5)	C24—N14—C21—N13	−180.0 (3)
C8—N8—C7—N6	−179.5 (3)	C24—N14—C21—C22	0.4 (4)
C9—N8—C7—C10	175.3 (3)	C20—N13—C21—N14	−1.5 (5)
C8—N8—C7—C10	1.6 (5)	C19—N13—C21—N14	176.5 (3)
C12—N6—C7—N8	176.8 (3)	C20—N13—C21—C22	178.1 (3)
C12—N6—C7—C10	−4.3 (4)	C19—N13—C21—C22	−3.9 (5)
N8—C7—C10—C11	−179.9 (3)	N14—C21—C22—C23	−0.3 (4)
N6—C7—C10—C11	1.2 (4)	N13—C21—C22—C23	−179.9 (3)
C12—N5—C11—C10	−0.3 (5)	C24—N15—C23—C22	1.0 (5)
C12—N5—C11—Cl4	179.4 (2)	C24—N15—C23—Cl2	−178.6 (2)
C7—C10—C11—N5	1.2 (5)	C21—C22—C23—N15	−0.4 (5)
C7—C10—C11—Cl4	−178.6 (2)	C21—C22—C23—Cl2	179.2 (2)
C7—N6—C12—N7	−175.9 (3)	C21—N14—C24—N16	178.2 (3)
C7—N6—C12—N5	5.5 (5)	C21—N14—C24—N15	0.3 (5)
C11—N5—C12—N6	−3.2 (5)	C23—N15—C24—N14	−1.0 (5)
C11—N5—C12—N7	178.2 (3)	C23—N15—C24—N16	−178.8 (3)

### Hydrogen-bond geometry (Å, º)

**Table:**

*D*—H···*A*	*D*—H	H···*A*	*D*···*A*	*D*—H···*A*
N11—H11*B*···N1	0.87	2.48	3.310 (4)	161
N3—H3*A*···N9	0.86	2.41	3.264 (4)	168
N16—H16*B*···N15^i^	0.87	2.17	3.038 (4)	174
N11—H11*A*···N10^ii^	0.87	2.21	3.086 (4)	174
N7—H7*B*···N5^iii^	0.88	2.19	3.064 (3)	178
N3—H3*B*···N6^iv^	0.89	2.35	3.230 (4)	169
N7—H7*A*···N2^iv^	0.87	2.33	3.136 (3)	155

Symmetry codes: (i) −*x*+2, −*y*+2, −*z*+2; (ii) −*x*, −*y*, −*z*+1; (iii) −*x*+1, −*y*+1, −*z*; (iv) −*x*+1, −*y*+1, −*z*+1.
